# Supplementary material for: Functional gene delivery to and across brain vasculature of systemic AAVs with endothelial-specific tropism in rodents and broad tropism in primates
Source: Nat Commun. 2023 Jun 8;14:3345. doi: 10.1038/s41467-023-38582-7 (PMC10250345; doi:10.1038/s41467-023-38582-7)
Supplement: Supplementary file 1 — Supplementary Information [file 41467_2023_38582_MOESM1_ESM.pdf]

Supplementary Figure 1: Detailed characterization of AAV9-X1 in the mouse brain.

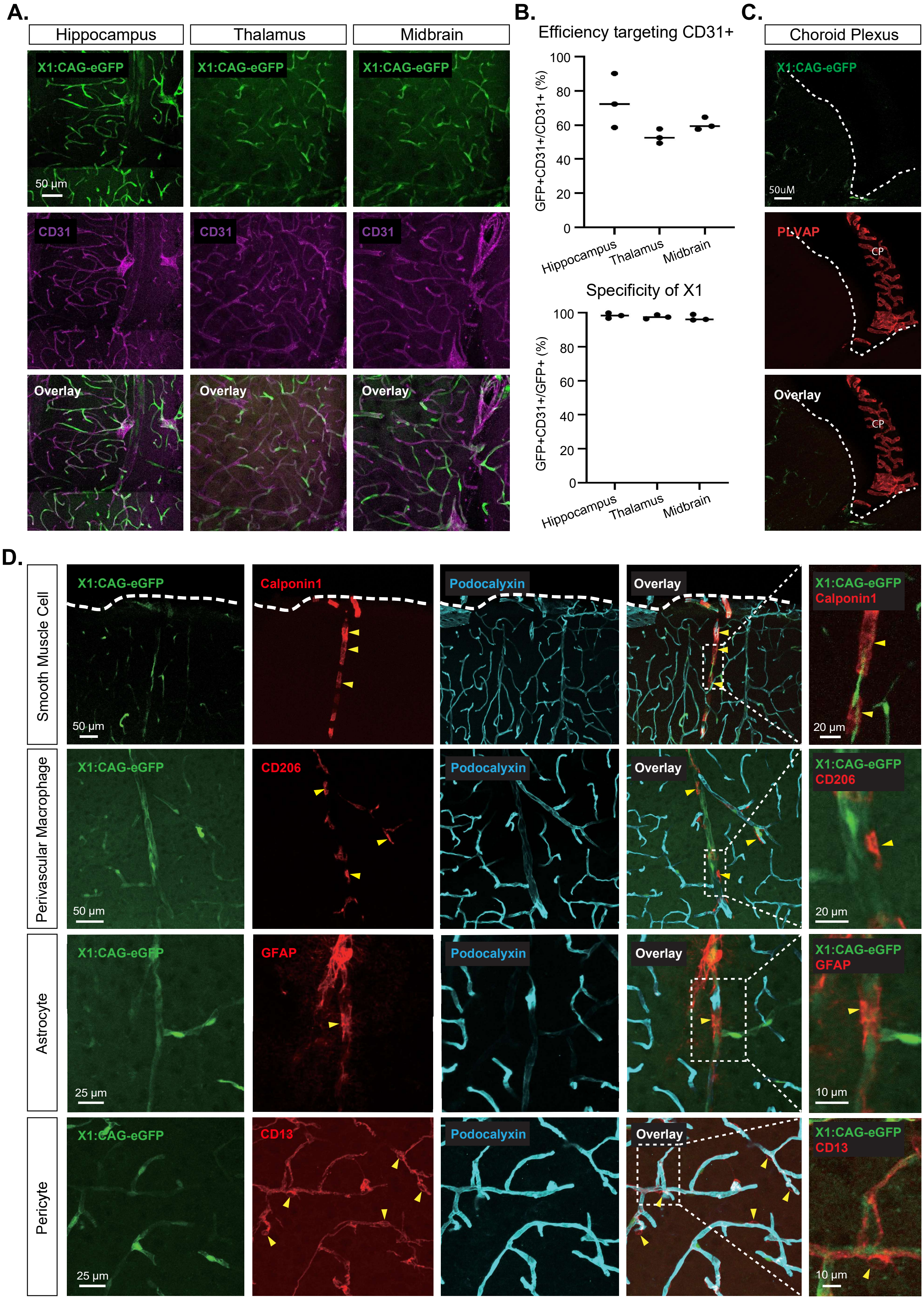

Supplementary Figure 1: Detailed characterization of AAV9-X1 in the mouse brain.

A. Representative images of AAV-X1 vector-mediated expression of eGFP (green) in the brain. The tissues were co-stained with GLUT1 marker (magenta) (scale bar: 50 µm). **B.** (Top) Percentage of AAV-mediated eGFP-expressing cells that overlap with GLUT1+ markers across brain regions, representing the efficiency of the vectors in targeting CD31+ cells. Each data point shows the mean ± SEM of 3 slices per mouse. (Bottom) Percentage of CD31+ markers in AAV-mediated eGFP-expressing cells across brain regions, representing the specificity of the vectors in targeting GLUT1+ cells. (n=4 per group, ~8 weeks old C57BL/6J males, $3x10^{11}$ vg IV dose per mouse, 3 weeks of expression). Source data are provided as a Source Data file. **C-D.** EGFP expression is seen only in endothelial cells possessing BBB characteristics, and not in other vascular cells. **C.** PLVAP (red)-positive endothelial cells in choroid plexus (CP) do not express eGFP. The ventricular border is indicated by the dashed line. N=3 mice were tested and examined. **D.** Representative images of brain sections co-stained with endothelial cell marker (podocalyxin, in cyan) and (in red, with yellow arrowheads) markers for smooth muscle cells (calponin 1), perivascular macrophages (CD206), astrocytes (GFAP), and pericytes (CD13). The dashed lines indicate the cortical surface. N=3 mice were tested and examined.

Supplementary Figure 2: Biodistribution of engineered AAVs across organs.

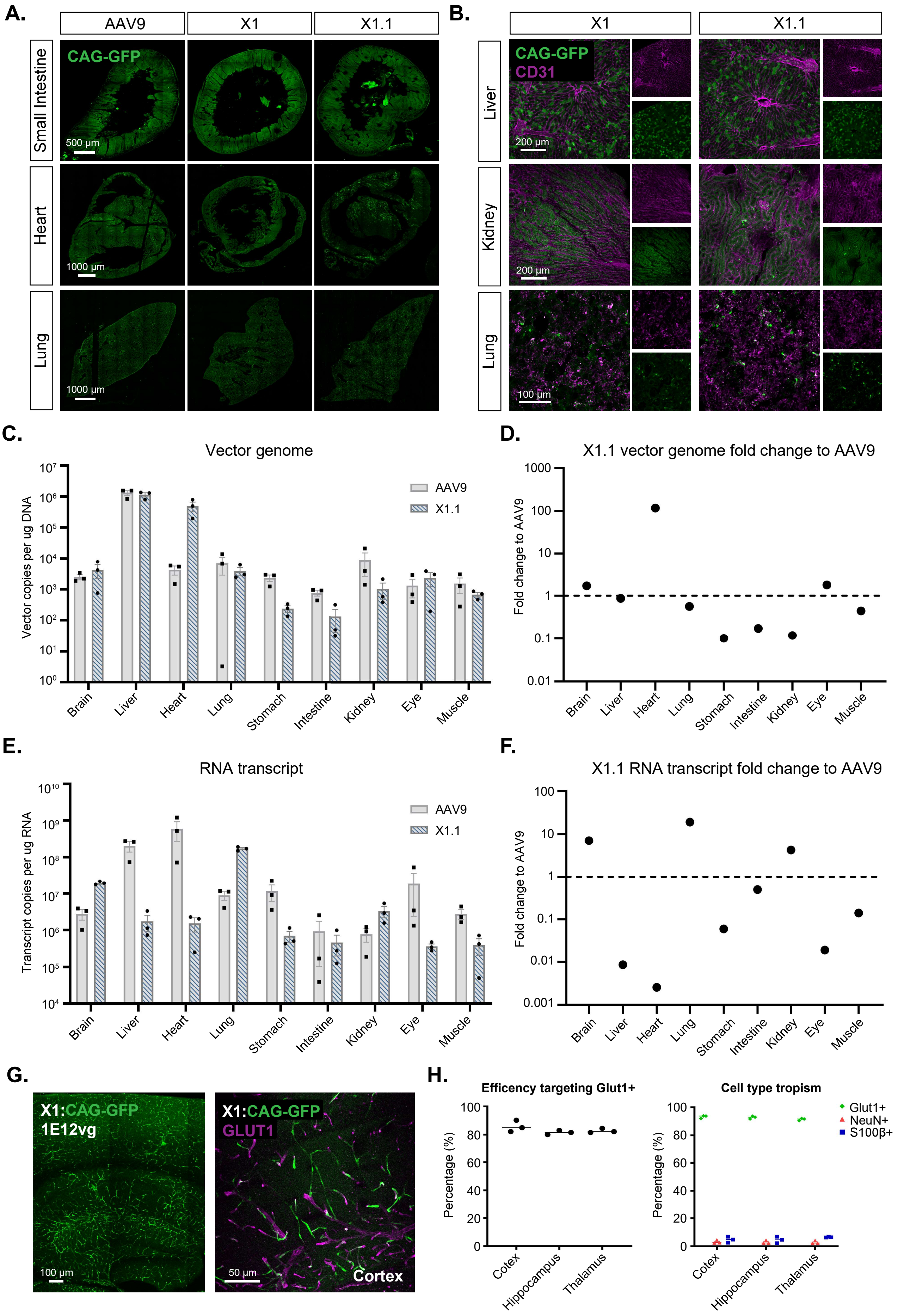

Supplementary Figure 2: Biodistribution of AAVs across mice organs.

A. Representative images of AAV9, AAV-X1, and AAV-X1.1 vector-mediated expression of eGFP in the small intestine (scale bar: 500 µm), heart (scale bar: 1000 µm) and lung (scale bar: 1000 µm) (n≥4 per group, ~8 week-old C57BL/6J males, 3E11 vg IV dose per mouse, 3 weeks of expression). **B.** Representative images of AAV-X1, and AAV-X1.1 vector-mediated expression of eGFP in the liver (scale bar: 500 µm), kidney (scale bar: 1000 µm) and lung (scale bar: 1000 µm) (n=4 per group, ~8 week-old C57BL/6J males, 3E11 vg IV dose per mouse, 3 weeks of expression). The tissues were co-stained with CD31 (magenta). **C**. Vector genome of AAV9 or AAV-X1 per ug DNA across organs in mice following I.V. delivery (n=3 per group, ~8 week-old C57BL/6J males, 3E11 vg IV dose per mouse, 3 weeks of expression). Data are presented as mean values +/- SEM. **D**. Vector genome of AAV-X1 fold change of vector genome of AAV9 across organs. (n=3 per group, ~8 week-old C57BL/6J males, 3E11 vg IV dose per mouse, 3 weeks of expression). **E**. RNA transcript of AAV9 or AAV-X1 per ug RNA across organs in mice following I.V. delivery, GADPH was used to normalize across organs (n=3 per group, ~8 week-old C57BL/6J males, 3E11 vg IV dose per mouse, 3 weeks of expression). Data are presented as mean values +/- SEM. **F**. RNA transcript of AAV-X1 fold change of RNA transcript of AAV9 across organs. (n=3 per group, ~8 week-old C57BL/6J males, 3E11 vg IV dose per mouse, 3 weeks of expression). Source data are provided as a Source Data file. **G.** AAV-X1 vector-mediated expression of eGFP in the cortex (n=3, ~8 week-old C57BL/6J males, 1E12 vg IV dose per mouse, 3 weeks of expression). The tissues were co-stained with GLUT1 (magenta). Quantification can be seen in **H**. (Left) Percentage of AAV-mediated eGFP-expressing cells that overlap with the GLUT1+ marker across brain regions, representing the efficiency of the vectors' targeting of GLUT1+ cells. (Right) Percentage of marker+ cells in AAV-mediated eGFP-expressing cells across brain regions, representing the specificity of the vectors. Each data point is the average of 3 slices of each mouse. Source data are provided as a Source Data file.

Supplementary Figure 3: Engineered AAVs are independent of Ly6a and show different expression patterns in pericyte-deficient mice.

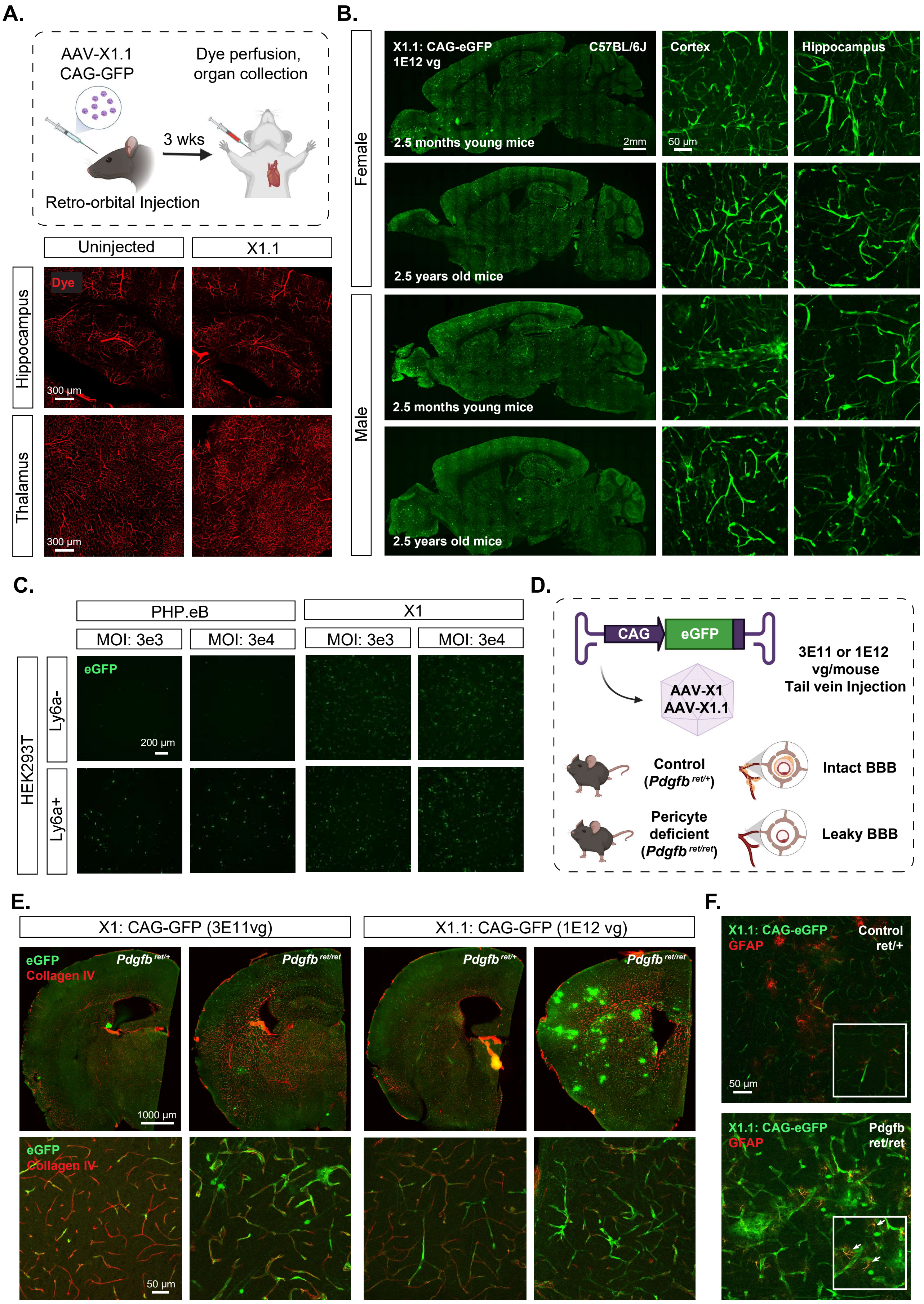

Supplementary Figure 3：Engineered AAVs are independent of Ly6a and show different expression patterns in pericyte-deficient mice.

A. (Top) Illustration of dye perfusion to evaluate the intactness of the BBB in AAV-injected mice. (Bottom) Representative images of dye staining (red) in hippocampus and thalamus (scale bars: 300 µm) (n=3 per group, 1E12 vg IV dose per mouse, 3 weeks of expression). **B.** Representative image of AAV-X1 vector-mediated expression of eGFP in the brains of both sexes and both young 2.5-month-old and aged 2.5-year-old C57BL/6J males (scale bars: 2 mm in whole brain and 50 µm in cortex/hippocampus) (n=3 per group, 1E12 vg IV dose per mouse, 3 weeks of expression). **C.** Representative images of AAV transduction in HEK293T cells with or without previous transfection of plasmids encoding Ly6a. (Packaged with ssAAV:CAG-eGFP, n= 3 per condition, 2-day expression, high dose: MOI 25000, low dose: MOI 2500). Scale bar: 200 µm. **D.** Illustration of AAV vector delivery to control mice and pericyte-deficient mice (Pdgfb ret/ret) for studying their transduction profile in BBB in different conditions (3E11 vg/mouse for X1, 1E12 vg/mouse for X1.1, female animals, tail vein injection, 3 weeks' expression). **E.** Representative images of AAV-mediated expression of eGFP (green) in coronal sections of mouse brain (scale bar: 1000 µm), and zoomed-in images of tissue co-stained with collagen IV marker (red) (scale bar: 2 mm). N=3 mice each condition. **F.** Representative images of tissue co-stained with GFAP marker (red) (scale bar: 50 µm). Boxes show further zoomed-in views of astrocytes that have endfeet on the vasculature; white arrows highlight the colocalization of eGFP expression and GFAP marker in Pdgfb ret/ret mice. N=3 mice each condition.

Supplementary Figure 4 : Hevin specificity and engineered AAVs efficently transduce human cells.

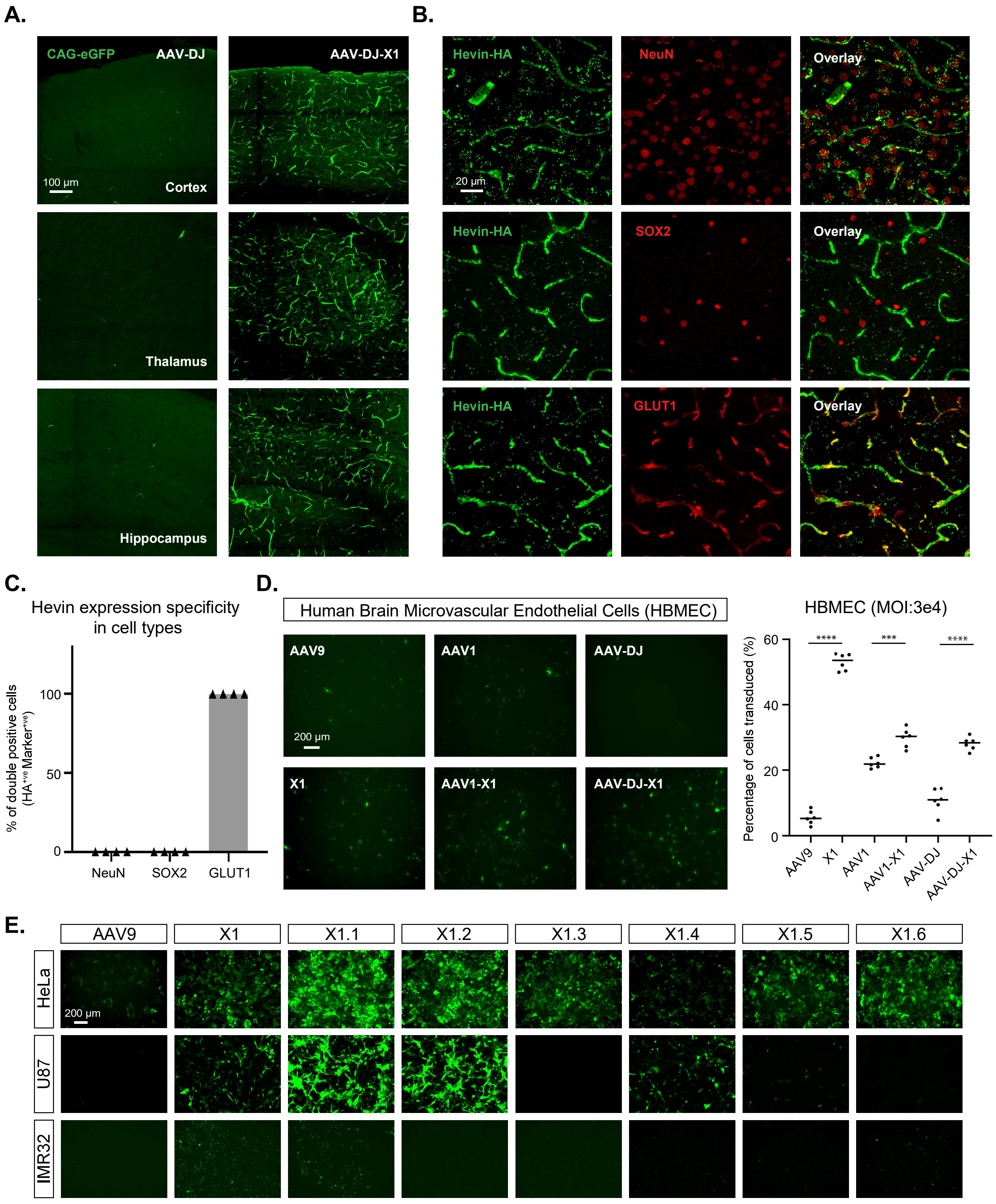

Supplementary Figure 4: Hevin specificity amd engineered AAVs efficiently transduce human cells.

A. Representative images of AAV-DJ and AAV-DJ-X1-mediated eGFP expression in the cortex, thalamus and hippocampus (scale bar: 100 µm) (C57BL/6J, n=3 per group, 3E11 vg IV dose per mouse, 3 weeks of expression). **B**. Representative images of AAV-X1.1 vector-mediated expression of Hevin in the brain. The tissues were co-stained with HA (green) and NeuN (red) or SOX2 (red) or GLUT1 (red) (scale bar: 30 µm). N=4 mice were tested. **C**. Quantification of AAV-X1.1-mediated Hevin expression across cell types, each data point is from one mouse. N=4 male mice were tested. Source data are provided as a Source Data file. **D**. (Left) Representative images of AAV (AAV9, X1, AAV1, AAV1-X1, AAV-DJ, AAV-DJ-X1)-mediated eGFP expression (green) in Human Brain Microvascular Endothelial Cells (HBMECs). (AAVs packaged with ssAAV:CAG-eGFP, n= 6 per condition, 1 day expression). (Right) Percentage of cells transduced by the AAVs, unpaired t tests are reported (****P=8e-12 for AAV9 versus X1, ***P=0.0002 for AAV1 versus AAV1-X1, ****P=1.3e-6 for AAV-DJ versus AAV-DJ-X1; n=6 per group, each data point is the mean of 3 technical replicates, mean ± SEM is plotted). Source data are provided as a Source Data file. **E.** Representative images of AAV (AAV9, X1, X1.1, X1.2, X1.3, X1.4, X1.5, X1.6)-mediated eGFP expression (green) in HeLa cells, U87 cells, and IMR32 cells (AAVs packaged with ssAAV:CAG-eGFP, n= 3 per condition, 1 day expression, MOI: 50000).

Supplementary Figure 5: Engineered AAVs efficiently transduce human cell lines and *ex vivo* macaque slices.

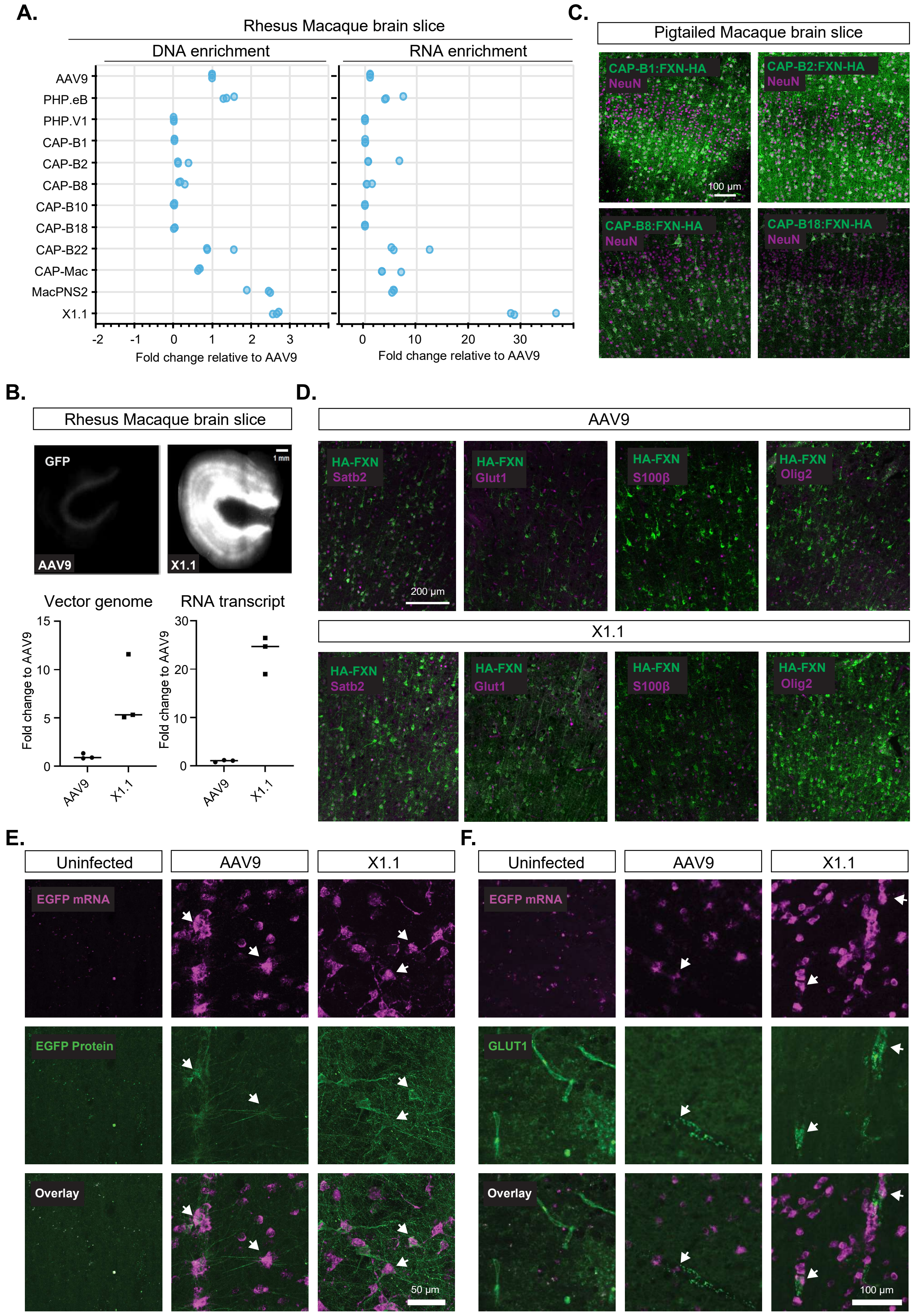

Supplementary Figure 5: Engineered AAVs efficiently transduce human cell lines and *ex vivo* macaque slices.

A. DNA and RNA level in rhesus macaque brain slices for AAVs, with DNA and RNA levels normalized to AAV9. N=3 independent slices from 1 rhesus macaque case are examined. Source data are provided as a Source Data file. **B.** (top) Representative images of AAV9 and X1.1-mediated CAG-GFP expression in *ex vivo* rhesus macaque brain slices. (bottom) Vector genome and RNA transcript of the AAVs in the slices, fold change to AAV9 was shown. N=3 independent slices from 1 rhesus macaque case are examined. Source data are provided as a Source Data file. **C.** Representative images of AAV (CAP-B1, CAP-B2, CAP-B8, CAP-B18)-mediated CAG-FXN-HA expression in *ex vivo* southern pig-tailed macaque brain slices. The tissues were co-stained with antibodies against HA (green) and NeuN (magenta). N=12 independent slices were examined over 3 pigtailed macaque cases. **D.** Representative images of AAV9 and X1.1-mediated CAG-FXN-HA expression in *ex vivo* southern pig-tailed macaque brain slices. The tissues were co-stained with antibodies against HA (green) and Satb2, GLUT1, S100β or Olig2 (magenta). N=12 independent slices were examined over 3 pigtailed macaque cases. **E, F.** mFISH analysis of EGFP expression in transduced cultured slices. Organotypic cortical slices were cultured from macaque brain, and were uninfected or infected by X1.1 or AAV9 encapsidated EGFP reporter vector. After eight days in culture, EGFP mRNA was resolved against EGFP protein (E) or GLUT-1 protein (F) in cortical white matter regions. Minimal background EGFP mRNA or protein was detected in the absence of virus (uninfected), EGFP mRNA positive cells often express EGFP protein (arrows, E), and EGFP mRNA positive cells are often found lining GLUT-1+ blood vessels (putative endothelial cells, or pericytes, arrows, F). Images are from stitched montages. N=3 independent slices were examined over 1 rhesus macaque case.

Supplementary Figure 6: Engineered AAV transduces the central nervous system in marmoset similarly to AAV9.

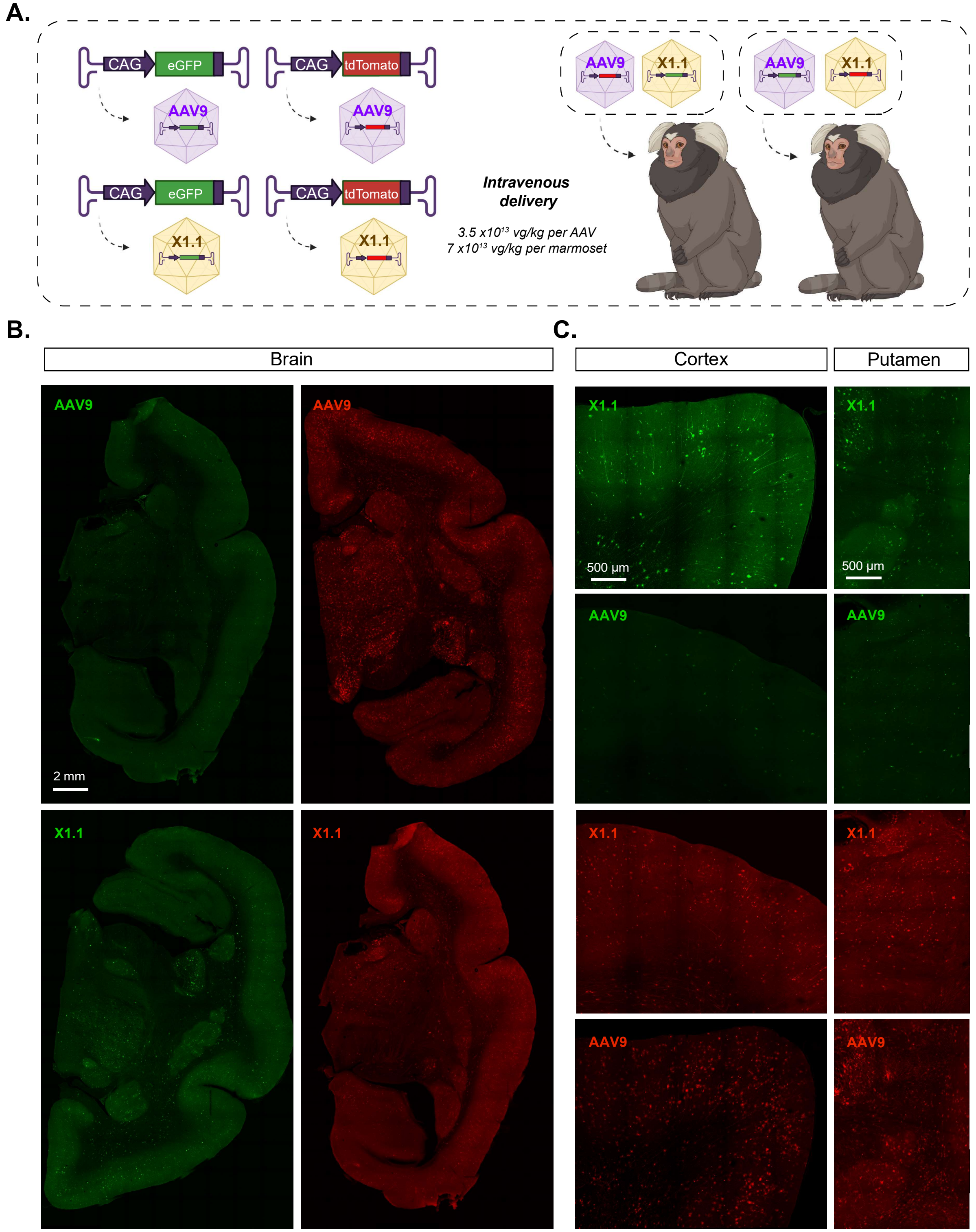

Supplementary Figure 6: Engineered AAV transduces the central nervous system in marmoset similarly to AAV9.

A. Illustration of AAV vector delivery to adult marmoset to study transduction across the CNS after 3 weeks of expression. The capsids (AAV9/X1.1) and their corresponding genomes (ssAAV:CAG-eGFP/tdTomato) are shown on the left. Two AAV vectors packaged with different colored fluorescent reporters were mixed and intravenously delivered at a total dose of $7x10^{13}$ vg/kg per adult marmoset (16 month-old *Callithrix jacchus*, i.e. $3.5x10^{13}$ vg/kg per AAV, 1 female animal and 1 male animal). Representative images of **B.**, coronal brain sections of the midbrain (scale bar: 2 mm), and **C.**, select brain areas: cortex and putamen (scale bar: 500 µm), showing AAV9 vector-mediated expression of eGFP (green) or tdTomato (red), X1.1-mediated expression of eGFP (green) and X1.1-mediated expression of tdTomato (red).

Supplementary Figure 7: Biodistribution of engineered AAV in rhesus macaque following systemic delivery

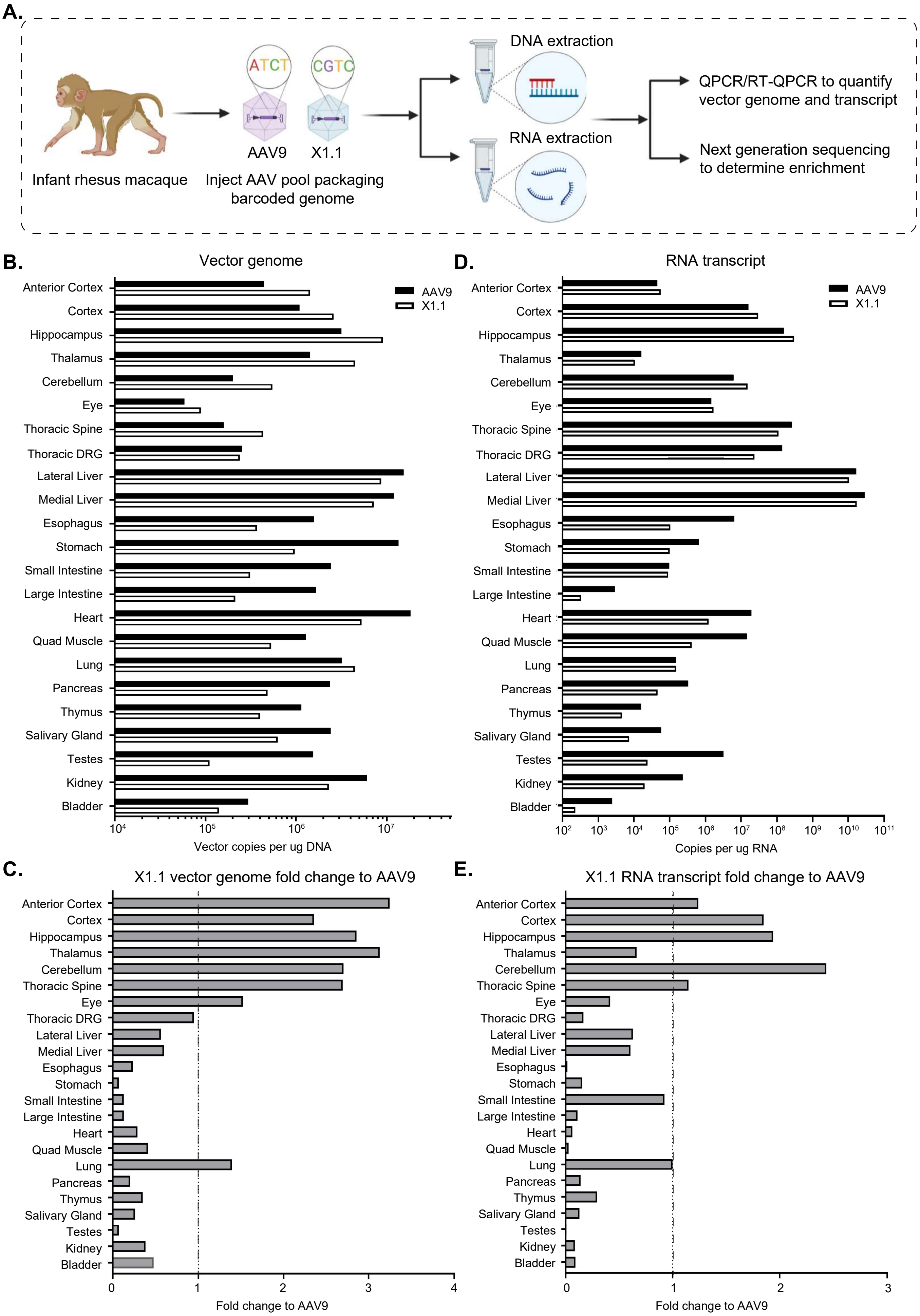

Supplementary Figure 7: Biodistribution of engineered AAV in rhesus macaque following systemic delivery.

A. Illustration of AAV vector delivery to rhesus macaque to study biodistribution after 4 weeks of expression. The capsids (AAV9/X1.1) packaged with CAG-GFP genome containing a unique barcode were mixed and intravenously injected at a dose of $2x10^{13}$ vg/kg per macaque (*Macaca mulatta*, male, injected within 10 days of birth, male, i.e. $2x10^{13}$ vg/kg per AAV). After 4 weeks of expression, tissues were collected and DNA/RNA extractions were performed. Then QPCR/RT-QPCR were performed to quantify vector genome and transcript. Next generation sequencing was performed to determine enrichment. **B.** Vector genome of AAV9 or AAV-X1 per ug DNA across organs in rhesus macaque following IV delivery. **C**. Vector genome of AAV-X1 fold change of vector genome of AAV9 across organs in rhesus macaque. **D**. RNA transcript of AAV9 or AAV-X1 per ug RNA across organs in rhesus macaque following I.V. delivery, GADPH was used to normalize across organs. **E**. RNA transcript of AAV-X1 fold change of RNA transcript of AAV9 across organs. Source data are provided as a Source Data file.

Supplementary Table 1: Summary of engineering approaches for the novel AAVs.

Capsid	Backbone	Engineering
X1	AAV9	Insertion at 588/589 site of AAV9, GNNTRSV
X1.1	AAV9	Insertion at 588/589 site of AAV9, GNNTRSV; Substitution at 452-458 site of AAV9, DGAATKN
X1.2	AAV9	Insertion at 588/589 site of AAV9, GNNTRSV; Substitution at 452-458 site of AAV9, DGQSSKS
X1.3	AAV9	Insertion at 588/589 site of AAV9, GNNTRSV; Substitution at 452-458 site of AAV9, LQTSSPG
X1.4	AAV9	Insertion at 588/589 site of AAV9, GNNTRSV; 272 site of AAV9 mutate to A
X1.5	AAV9	Insertion at 588/589 site of AAV9, GNNTRSV; 503 site of AAV9 mutate to A
X1.6	AAV9	Insertion at 588/589 site of AAV9, GNNTRSV; 386 site of AAV9 mutate to A
AAV1-X1	AAV1	Insertion at 588/589 site of AAV1, GNNTRSV
AAV-DJ-X1	AAV-DJ	Insertion at 589/590 site of AAV-DJ, GNNTRSV

Supplementary Table 2: Summary of novel AAVs' performance across models tested.

Capsid	Model tested	Sex and age	Administration	Tropism
X1	Wildtype mice (C57BL/6J, BALB/CJ, FVB/NJ, CBA/J)	Both male and female, 6-8 weeks	Intravenously	Brain endothelial cell specific expression
	Transgenic mice (PDGFB-retention motif knock out mice)	Both male and female, 6-8 weeks	Intravenously	Brain endothelial cell specific expression in PDGFB ret/+. Endothelial, neuron, astrocyte expression in PDGFP ret/ret
	Human Brain Microvascular Endothelial cells	-	Direct application	Stronger expression comparing to controls including AAV9, AAV1, AAV2 etc.
X1.1	Wildtype mice (C57BL/6J)	Both male and female, 6-8 weeks or 2 years old	Intravenously	Brain endothelial cell specific expression
	Transgenic mice (Hevin-KO)	Male, 6-8 weeks	Intravenously	Brain endothelial cell specific expression
	Transgenic mice (PDGFB-retention motif knock out mice)	Both male and female, 6-8 weeks	Intravenously	Brain endothelial cell specific expression in PDGFB ret/+, endothelial, neuron, astrocyte expression in PDGFP ret/ret
	Rat	Both male and female, 6-8 weeks	Intravenously	Brain endothelial cell specific expression
	Marmoset	Both male and female, 14 months	Intravenously	Neuron, astrocyte and endothelial cell expression
	Rhesus Macaque	Both male and female, infant	Intravenously	Neuron and endothelial cell expression
	Macaque Brain Slice	Both male and female, adult 2~4 years,	Direct application	predominantly neuron expression with lesser astrocyte, oligodendrocyte, and endothelial cell expression
	Human Brain Microvascular Endothelial cells	-	Direct application	Stronger expression comparing to controls including AAV9, AAV1, AAV2 etc.
	Human Brain Slice		Direct application	Stronger expression comparing to controls including AAV9
