# Supplementary material for: Functional gene delivery to and across brain vasculature of systemic AAVs with endothelial-specific tropism in rodents and broad tropism in primates
Source: Nat Commun. 2023 Jun 8;14:3345. doi: 10.1038/s41467-023-38582-7 (PMC10250345; doi:10.1038/s41467-023-38582-7)
Supplement: Supplementary file 3 — Description of Additional Supplementary Files [file 41467_2023_38582_MOESM3_ESM.pdf]

**Description of Additional Supplementary Files**

File Name: Supplementary Movie 1
Description: **Macaque hindbrain injected with AAV9 and AAV9-X1.1.**
AAV9 packaging ssAAV:CAG-eGFP and AAV9-X1.1 packaging ssAAV:CAG-tdTomato were mixed and intravenously injected at a dose of 5x1013 vg/kg per macaque (Macaca mulatta, injected within 10 days of birth, female, i.e. 2.5x1013 vg/kg per AAV). Representative overview of macaque hindbrain.
