# Supplementary material for: Functional gene delivery to and across brain vasculature of systemic AAVs with endothelial-specific tropism in rodents and broad tropism in primates
Source: Nat Commun. 2023 Jun 8;14:3345. doi: 10.1038/s41467-023-38582-7 (PMC10250345; doi:10.1038/s41467-023-38582-7)
Supplement: Supplementary file 5 — Reporting Summary [file 41467_2023_38582_MOESM5_ESM.pdf]

nature portfolio

Corresponding author(s): Viviana Gradinaru

Last updated by author(s): Mar 14, 2023

Reporting Summary

Nature Portfolio wishes to improve the reproducibility of the work that we publish. This form provides structure for consistency and transparency in reporting. For further information on Nature Portfolio policies, see our Editorial Policies and the Editorial Policy Checklist.

Statistics

For all statistical analyses, confirm that the following items are present in the figure legend, table legend, main text, or Methods section.

n/a	Confirmed	
☐	☒	The exact sample size (*n*) for each experimental group/condition, given as a discrete number and unit of measurement
☐	☒	A statement on whether measurements were taken from distinct samples or whether the same sample was measured repeatedly
☐	☒	The statistical test(s) used AND whether they are one- or two-sided<br>*Only common tests should be described solely by name; describe more complex techniques in the Methods section.*
☒	☐	A description of all covariates tested
☒	☐	A description of any assumptions or corrections, such as tests of normality and adjustment for multiple comparisons
☐	☒	A full description of the statistical parameters including central tendency (e.g. means) or other basic estimates (e.g. regression coefficient) AND variation (e.g. standard deviation) or associated estimates of uncertainty (e.g. confidence intervals)
☐	☒	For null hypothesis testing, the test statistic (e.g. *F*, *t*, *r*) with confidence intervals, effect sizes, degrees of freedom and *P* value noted<br>*Give P values as exact values whenever suitable.*
☒	☐	For Bayesian analysis, information on the choice of priors and Markov chain Monte Carlo settings
☒	☐	For hierarchical and complex designs, identification of the appropriate level for tests and full reporting of outcomes
☒	☐	Estimates of effect sizes (e.g. Cohen's *d*, Pearson's *r*), indicating how they were calculated

Our web collection on statistics for biologists contains articles on many of the points above.

Software and code

Policy information about availability of computer code

Data collection	For images acquiring and processing, we used Zen Black 2.3 SP1, BZ-X Analyzer 1.4.0.1 and Imaris 9 software.
Data analysis	Statistical analysis was performed with Graph-Pad Prism 9.0.1 software. The codes used for M-CREATE data analysis are available on GitHub: https://github.com/GradinaruLab/mCREATE.

For manuscripts utilizing custom algorithms or software that are central to the research but not yet described in published literature, software must be made available to editors and reviewers. We strongly encourage code deposition in a community repository (e.g. GitHub). See the Nature Portfolio guidelines for submitting code & software for further information.

Data

Policy information about availability of data

All manuscripts must include a data availability statement. This statement should provide the following information, where applicable:

- Accession codes, unique identifiers, or web links for publicly available datasets
- A description of any restrictions on data availability
- For clinical datasets or third party data, please ensure that the statement adheres to our policy

All sequences of AAV variants developed in this study are provided in materials and methods. Key vector plasmid AAV-X1.1 is available at Addgene (#196836). All other constructs and tools will be available through the Beckman Institute CLOVER Center (https://clover.caltech.edu/). Source data are provided with this paper. Other data that support the findings of this study are available from the corresponding author upon request.

Human research participants

Policy information about studies involving human research participants and Sex and Gender in Research.

Reporting on sex and gender	Recruitment of the patients is performed outside of the supervision of the Allen Institute by a hospital-appointed case coordinator under the authority of the IRB of the participating hospital. Brain tissue were collected based on availability, both female and male tissue were used in this study.
Population characteristics	Human brain tissue came from peri-tumor tissue removed from the right parietal cortex of a 55-year-old female patient and the right frontal cortex of a 61-year-old male patient to access deep brain tumors.
Recruitment	Recruitment of the patients is performed outside of the supervision of the Allen Institute by a hospital-appointed case coordinator under the authority of the IRB of the participating hospital.
Ethics oversight	All human neurosurgical tissue studies were approved by the Western Institutional Review Board. Human neurosurgical specimens were obtained with informed consent of patients that underwent neocortex resection for the treatment of temporal lobe epilepsy or for tumor removal. Specimens for research were not required for diagnostic purposes and were distal to the pathological focus of the surgical resections.

Note that full information on the approval of the study protocol must also be provided in the manuscript.

Field-specific reporting

Please select the one below that is the best fit for your research. If you are not sure, read the appropriate sections before making your selection.

[x] Life sciences [ ] Behavioural & social sciences [ ] Ecological, evolutionary & environmental sciences

For a reference copy of the document with all sections, see nature.com/documents/nr-reporting-summary-flat.pdf

Life sciences study design

All studies must disclose on these points even when the disclosure is negative.

Sample size	No sample size calculation was performed.
Data exclusions	No data were excluded from the analysis.
Replication	Each in vitro experiment were evaluated in multiple repeats and showed similar result. Each animal experiment used two or more animals except the macaque experiment. We observed good correlation between the 2 independent macaque experiment we performed. We also observed similar results between repeats of other animal experiment.
Randomization	Samples or animals were randomly allocated into experimental group. For imaging we randomly selected the fields-of-views in the specific regions of the brain.
Blinding	The investigators were not blinded to allocation during experiments. For outcome assessment such as image collection and analysis, we used a barcode for each condition and matched them after analysis.

Reporting for specific materials, systems and methods

We require information from authors about some types of materials, experimental systems and methods used in many studies. Here, indicate whether each material, system or method listed is relevant to your study. If you are not sure if a list item applies to your research, read the appropriate section before selecting a response.

Materials & experimental systems

n/a	Involved in the study	
[ ]	[x]	Antibodies
[ ]	[x]	Eukaryotic cell lines
[x]	[ ]	Palaeontology and archaeology
[ ]	[x]	Animals and other organisms
[x]	[ ]	Clinical data
[x]	[ ]	Dual use research of concern

Methods

n/a	Involved in the study	
[x]	[ ]	ChIP-seq
[x]	[ ]	Flow cytometry
[x]	[ ]	MRI-based neuroimaging

Antibodies

Antibodies used	Anti-CD31 from Histonova ,Cat # DIA-310, diluted 1:100<br>Anti-podocalyxin from R&D Systems, Cat # AF1556, diluted 1:100<br>Anti-CD13 from AbD Serotec, Cat # MCA2183EL, diluted 1:100<br>Anti-CNN-1 (Calponin 1) from Abcam, Cat # Ab46794, diluted 1:100<br>Anti-Collagen-IV, from AbD Serotec, Cat # 2150-1470, diluted 1:300<br>Anti-GFAP from Invitrogen, Cat # 13-0300, diluted 1:600<br>Anti-HA from Roche, Cat# 11867423001, diluted 1:200<br>Anti-GFP from Aves Labs, Cat# GFP-1020, diluted 1:500<br>Anti-GLUT1 from Millipore, Cat# 07-1401, diluted: 1:500<br>Anti-S100β from Abcam, Cat# ab52642, diluted: 1:500,<br>Anti-NeuN from Abcam, Cat# ab177487, diluted: 1:500<br>Anti-VgluT2 from Synaptic System, Cat# 135 404, diluted: 1:500<br>Anti-PSD95 from Thermo Fisher, Cat# 51-6900, diluted: 1:500<br>Anti-HA from Biolegend, Cat# 901513, diluted: 1:1000<br>Anti-NeuN from Millipore, Cat# ABN78, diluted: 1:1000<br>Anti-Olig2 from Abcam, Cat# AB9610, diluted 1:1000<br>Anti-S100β from Millipore, Cat# S2532, diluted 1:1000<br>Anti-mouse Alexa488 from Invitrogen, Cat# A-11001, diluted 1:1000<br>Anti-mouse Alexa555 from Invitrogen, Cat# A-21422, diluted 1:1000<br>Anti-rabbit Alexa555 from Invitrogen, Cat# A-21428, diluted 1:1000<br>Anti-rabbit Alexa647 from Invitrogen, Cat# A-21245, diluted 1:1000<br>Anti-rat Alexa488 from Invitrogen, Cat# A-11006, diluted 1:1000<br>Anti-rat Alexa555 from Invitrogen, Cat# A-21434, diluted 1:1000<br>Anti-rabbit AF594 from Jackson Immuno, Cat# 711-585-152, diluted 1:1000
Validation	Anti-CD31 from Histonova ,Cat # DIA-310 (https://www.dianova.com/en/shop/dia-310-anti-cd31-mssw-from-rat-sz31-unconj-for-mouse-ffpe-tissue/)<br>Anti-podocalyxin from R&D Systems, Cat # AF1556 (https://www.rndsystems.com/products/mouse-podocalyxin-antibody_af1556)<br>Anti-CD13 from AbD Serotec, Cat # MCA2183EL (https://www.bio-rad-antibodies.com/monoclonal/mouse-cd13-antibody-r3-63-mca2183.html?f=purified)<br>Anti-CNN-1 (Calponin 1) from Abcam, Cat # Ab46794 (https://www.abcam.com/products/primary-antibodies/calponin-1-antibody-ep798y-ab46794.html)<br>Anti-Collagen-IV, from AbD Serotec, Cat # 2150-1470 (https://www.bio-rad-antibodies.com/polyclonal/mouse-collagen-iv-antibody-2150-1470.html?f=purified)<br>Anti-GFAP from Invitrogen, Cat # 13-0300 (https://www.thermofisher.com/antibody/product/GFAP-Antibody-clone-2-2B10-Monoclonal/13-0300)<br>Anti-HA from Roche, Cat# 11867423001 (https://www.sigmaaldrich.com/US/en/product/roche/roahaha)<br>Anti-GFP from Aves Labs, Cat# GFP-1020 (https://www.aveslabs.com/products/anti-green-fluorescent-protein-antibody-gfp)<br>Anti-GLUT1 from Millipore, Cat# 07-1401 (https://www.emdmillipore.com/US/en/product/Anti-GLUT-1-Antibody-CT,MM_NF-07-1401?ReferrerURL=https%3A%2F%2Fwww.google.com%2F)<br>Anti-S100β from Abcam, Cat# ab52642 (https://www.abcam.com/products/primary-antibodies/s100-beta-antibody-ep1576y-astrocyte-marker-ab52642.html)<br>Anti-NeuN from Abcam, Cat# ab177487 (https://www.abcam.com/products/primary-antibodies/neun-antibody-epr12763-neuronal-marker-ab177487.html)<br>Anti-VgluT2 from Synaptic System, Cat# 135 404 (https://sysy.com/product/135404)<br>Anti-PSD95 from Thermo Fisher, Cat# 51-6900 (https://www.thermofisher.com/antibody/product/PSD-95-Antibody-Polyclonal/51-6900)<br>Anti-HA from Biolegend, Cat# 901513 (https://www.biolegend.com/en-us/products/anti-ha-11-epitope-tag-antibody-11071?GroupID=GROUP26)<br>Anti-NeuN from Millipore, Cat# ABN78 (https://www.emdmillipore.com/US/en/product/Anti-NeuN-Antibody-rabbit,MM_NF-ABN78)<br>Anti-Olig2 from Abcam, Cat# AB9610 (https://www.emdmillipore.com/US/en/product/Anti-Olig-2-Antibody,MM_NF-AB9610)<br>Anti-S100β from Millipore, Cat# S2532 (https://www.sigmaaldrich.com/US/en/product/sigma/s2532)<br>Anti-mouse Alexa488 from Invitrogen, Cat# A-11001 (https://www.thermofisher.com/antibody/product/Goat-anti-Mouse-IgG-H-L-Cross-Adsorbed-Secondary-Antibody-Polyclonal/A-11001)<br>Anti-mouse Alexa555 from Invitrogen, Cat# A-21422 (https://www.thermofisher.com/antibody/product/Goat-anti-Mouse-IgG-H-L-Cross-Adsorbed-Secondary-Antibody-Polyclonal/A-21422)<br>Anti-rabbit Alexa555 from Invitrogen, Cat# A-21428 (https://www.thermofisher.com/antibody/product/Goat-anti-Rabbit-IgG-H-L-Cross-Adsorbed-Secondary-Antibody-Polyclonal/A-21428)<br>Anti-rabbit Alexa647 from Invitrogen, Cat# A-21245 (https://www.thermofisher.com/antibody/product/Goat-anti-Rabbit-IgG-H-L-Highly-Cross-Adsorbed-Secondary-Antibody-Polyclonal/A-21245)<br>Anti-rat Alexa488 from Invitrogen, Cat# A-11006 (https://www.thermofisher.com/antibody/product/Goat-anti-Rat-IgG-H-L-Cross-Adsorbed-Secondary-Antibody-Polyclonal/A-11006)<br>Anti-rat Alexa555 from Invitrogen, Cat# A-21434 (https://www.thermofisher.com/antibody/product/Goat-anti-Rat-IgG-H-L-Cross-Adsorbed-Secondary-Antibody-Polyclonal/A-21434)<br>Anti-rabbit AF594 from Jackson Immuno, Cat# 711-585-152 (https://www.jacksonimmuno.com/catalog/products/711-585-152)

Eukaryotic cell lines

Policy information about cell lines and Sex and Gender in Research

Cell line source(s)	HEK293T cells (ATCC, CRL-3216)<br>HeLa cells (ATCC, CCL-2)<br>U87 cells (ATCC, HTB-14)<br>IMR32 cells (ATCC, CCL-127)<br>Human brain microvascular endothelial cells (ScienCell Research Laboratories, cat. no. 1000)<br>Expi293F cells (Thermo Fisher, Cat# A14527)
Authentication	None of the cell lines used were authenticated.
Mycoplasma contamination	The cell lines were not tested for mycoplasma contamination.
Commonly misidentified lines (See ICLAC register)	No cell lines used in this studies are commonly misidentified.

Animals and other research organisms

Policy information about studies involving animals; ARRIVE guidelines recommended for reporting animal research, and Sex and Gender in Research

Laboratory animals	For mice studies, we purchased C57BL/6J (000664), BALB/cJ (000651), FVB/NJ (001800), CBA/J (000656) and Tek-Cre (008863) mice (both male and females, 6-8 weeks or 2 years old) from the Jackson laboratory. 4-month-old male Hevin KO mice (Kucukdereli et al. 2011) were injected at Duke University. 4-5-month-old female Pdgfb ret/ret mice (Lindblom et al. 2003) were injected at University of Zurich. For the rat experiment, female rats were injected at UCL. For marmoset experiment, 16-month-old marmosets (1 female and 1 male) were used at UCSD. For macaque experiment, 1 female infant macaque and 1 male infant macaque were used, both were injected within 10 days of birth.
Wild animals	The study did not involve wild animals.
Reporting on sex	Both sexes were used across the whole study and the sex information was indicated in the detailed descriptions for each experiments. No obvious sex difference were observed in our side by side experiments with both sexes as shown in Supplementary Figure 3B.
Field-collected samples	The study did not involve field-collected samples.
Ethics oversight	All animal procedures in mice that were carried out in this study were approved by the California Institute of Technology Institutional Animal Care and Use Committee (IACUC), Caltech Office of Laboratory Animal Resources (OLAR), Cantonal Veterinary Office Zurich (license number ZH194/2020, 32869/2020), Duke Division of Laboratory Animal Resources (DLAR).<br>All experimental procedures in rats were conducted at UCL according to the UK Animals Scientific Procedures Act (1986) and under personal and project licenses granted by the Home Office following appropriate ethics review.<br>All experimental procedures performed on marmosets were approved by the University of California, San Diego, Institutional Animal Care and Use Committee (IACUC) and in accordance with National Institutes of Health and the American Veterinary Medical Association guidelines. Two female animals and one male animal were used in this study and received intravenous injections of AAVs.<br>All experimental procedures performed on rhesus macaques were approved by the International Animal Care and Use Committee at the University of California, Davis and the California National Primate Research Center (CNPRC). One infant female animal and one infant male animal was used in this study and received intravenous injections of AAVs.

Note that full information on the approval of the study protocol must also be provided in the manuscript.
